# Effects of tobacco addiction on links between early life adversities, sleep disturbance, and depression: A moderated mediation approach

**DOI:** 10.1016/j.pmedr.2020.101225

**Published:** 2020-10-20

**Authors:** Arwa Ben Salah, Motohiro Nakajima, Briana N DeAngelis, Mustafa al'Absi

**Affiliations:** a Department of Community Medicine, Faculty of Medicine of Monastir, University of Monastir, Monastir, Tunisia; b Family Medicine and Biobehavioral Health, University of Minnesota Medical School, Duluth, MN, United States

**Keywords:** Tobacco use, Addiction, Sleep quality, Early life adversities, Depression

## Abstract

Despite the well-established relationship between early life adversities (ELA) and depression, the underlying mechanisms for this link remain less clear and need to be developed. The aim of this study was to advance our understanding of this link by testing the mediating role of sleep disturbances and the moderating role of tobacco use in this mediation. A total of 579 smokers and non-smokers were recruited in two US communities (Duluth and Minneapolis, MN). Simple and moderated mediation analyses were performed using the PROCESS macro for SPSS, with the number of ELA as an independent variable, depression symptoms assessed by the Patient Health Questionnaire-9 (PHQ-9) as a dependent variable, sleep quality assessed by the Pittsburgh Sleep Quality Index (PSQI) as a mediator, and smoking status as a moderator variable. The study demonstrated that ELA and depressive symptoms were positively correlated; and sleep quality fully mediated this relationship. This mediation was moderated by tobacco use (index of moderated mediation = 0.10, 95%CI [0.03; 0.19]) and was more pronounced among smokers (b = 0.14, 95%CI [0.07; 0.23]) than non-smokers (b = 0.04, 95%CI [0.0002; 0.10]). Subsequent mediation analyses run separately for each component of the PSQI suggested that individuals who experienced ELA and who were smokers had greater delays in sleep onset and were more likely to sleep for a shorter duration, both of which predicted greater depressive symptoms. Sleep quality is therefore a promising ELA-related target for preventive and therapeutic interventions as well as for further research in depression and tobacco addiction.

## 1 Background

Depression is a global public health concern that is pervasive in the world; and in the U.S.A., it affects 7.6% of persons aged 12 or over (Pratt and Brody, 2014). It is associated with long-term morbidity and with substantial economic burden; and depression is predicted to become the world’s leading cause of disability-adjusted life –years by 2030 (Sarah-Jayne, 2019). As a consequence, there has been growing interest in identifying at-risk populations, risk factors, and underlying mechanisms in order to guide preventive and therapeutic strategies for depression. In this study, we investigate sleep quality as a potential mechanism linking early life adversities (ELA) with increased risk of depression, with a special focus on understanding the potential moderating role of tobacco use in these relationships.

### 1.1 ELA and depressive symptoms

ELA and childhood trauma (CT) constitute risk factors for multiple physical and mental health problems throughout the life course (Dube et al., 2003, Brown et al., 2009, Nelson et al., 2017, Duffy et al., 2018). Their relationships with depression, anxiety, and other psychiatric problems are well established (Chang et al., 2019, Heim and Nemeroff, 2001, Pirkola et al., 2005, Li et al., 2016); and several studies have demonstrated that the number, specific nature (type), timing, and other characteristics of adversities are linked to chronicity of depression, severity of its symptoms, and treatment response (Nelson et al., 2017, Schalinski et al., 2016, Gilbert et al., 2009, Mandelli et al., 2015). What remains less clear, however, are mediators and moderators of these relationships. Identifying key factors implicated in the relationships between ELA/CT and mental health outcomes is important for more effective and well-targeted health interventions to prevent and treat depression across the life course.

### 1.2 Sleep as a potential mediator of ELA and depression

Sleep disturbances and poor sleep quality are also prevalent health concerns that affect 50 to 70 million Americans (Colten and Altevogt, 2006); and they represent one mechanism through which ELA may contribute to risk for mental health problems. Evidence indicates that ELA are related to increased risk of a wide range of self-reported sleep disorders and disturbances, such as nightmare frequency and distress, sleep apnea, and narcolepsy (Kajeepeta et al., 2015, Brindle et al., 2018). Extant research also demonstrates that sleep disorder severity increases as the number of ELA increase (Koskenvuo et al., 2010, Chapman et al., 2011, Chapman et al., 2009).

Given recent evidence that suggests sleep disturbances may serve as an independent risk factor for depression (Baglioni et al., 2011, Liu et al., 2018, Fang et al., 2019), predicting its onset, persistence, and recurrence (Johnson et al., 2006, Perlis et al., 2006, Zhai et al., 2015, Benca and Peterson, 2008), it is plausible that sleep mediates the relationship between ELA and depression. Indeed, there is a growing body of research suggesting that sleep mediates the relationship between trauma and poor health outcomes through multiple mechanisms at biological, cognitive, psychosocial, and behavioral levels (Spilsbury, 2009). For instance, a study by Picchioni and colleagues (Picchioni et al., 2010) found that insomnia partially mediated the link between combat-related distress and depressive symptoms among veterans. Furthermore, a recent study found that sleep partially mediated the relationship between childhood trauma and depression (Jones et al., 2018), although this investigation focused on a very specific clinical population (methamphetamine-using men). Combined, these findings suggest that ELA may indirectly impact depression through sleep quality.

### 1.3 Tobacco use as a moderator of the relationships among ELA, sleep, and depression

Tobacco use is also related to sleep problems (Boakye et al., 2018, Liao et al., 2019) and poorer mental health and depression (Anda et al., 1990, Anda et al., 1999, Glassman et al., 1990, Boden et al., 2010, Fluharty et al., 2017, Breslau et al., 1998, Mathew et al., 2017, Taylor et al., 2014); and it is possible that chronic tobacco use moderates both the relationship between ELA and sleep problems as well as the relationship between sleep problems and depression. Tobacco products contain nicotine, which activates multiple pathways, including the central nervous system and the hypothalamic–pituitary–adrenal (HPA) axis; and evidence indicates that increased HPA activity is associated with impaired sleep quality (van Dalfsen and Markus, 2018). Thus, is possible that the physiological effects of chronic tobacco consumption attenuates the relationship between ELA and sleep problems. Numerous studies have also shown a positive relationship between tobacco use and mental illness, including depression and anxiety disorders (Fluharty et al., 2017); however, no studies have examined the potential role of tobacco use in moderating the relationship between sleep problems and depression.

### 1.4 Current study

In the current study, we addressed two hypotheses and one research question. First, we predicted a positive association between ELA and symptoms of depression. Second, we hypothesized that this association would be mediated by sleep quality. We also examined whether the indirect effect of ELA on depression would vary as a function of smoking status.

## 2 Methods

### 2.1 Participants

In this paper, we included a total of 579 participants pooled from three differently designed studies that examined psychobiological mechanisms of stress and tobacco addiction. The data reported here were based on common measures collected prior to starting each of these studies. Interested participants contacted the laboratory to complete a phone screening during which eligibility criteria were assessed. In this screening, participants were asked about the history of mental and physical disorders as well as tobacco use. Eligible participants had to be free from major physical illnesses (e.g., cardiac disease, hypertension, renal or hepatic disease, diabetes) and active psychiatric disorders (e.g., psychotic or bipolar disorders). Pregnant women were not eligible. A participant was considered a smoker if he or she had smoked at least 5 cigarettes per day for the past 2 years. Smoking was also biochemically verified (expired carbon monoxide levels). A participant was considered a non-smoker if he or she had not smoked for the last 5 years and had smoked<100 cigarettes in his or her lifetime. Nearly three-quarters (75.3%) were smokers. The imbalance between the number of smokers and non-smokers was due to the focus of the primary studies. The research was approved by the Institutional Review Board at the University of Minnesota.

### 2.2 Measures

#### 2.2.1 Depression symptoms:

Participants completed the Patient Health Questionnaire-9 (PHQ9), which is a nine-item self-report questionnaire that assesses the major symptoms of depression outlined in Diagnostic and Statistics Manual of Mental Disorders - Fifth Edition (Association and Association, 2013). The total score can range from 0 to 27 and can be used as a continuous measure, indicating severity of depression symptoms. The internal consistency for this measures was good as indicated by a Cronbach’s alpha of 0.86.

#### 2.2.2 Early life adversity:

To measure ELA, we used a modified version of a validated Adverse Childhood Experiences (ACEs) questionnaire (Felitti et al., 1998). Items related to 8 categories of ELA were included: verbal abuse (n = 2), physical abuse (n = 2), sexual abuse (n = 4), household member substance abuse (n = 2), household member mental illness (n = 1), mother treated violently (n = 4), parental separation (n = 1), and household member incarceration (n = 1). A participant was considered to be exposed to a category if he/she reported “yes” to one or more individual items within the category. The number of exposed ACE categories was summed within each participant to obtain an index of ELA (range 0–8: 0 = no ACE exposure; 8 = exposed to all ACE categories) (Anda et al., 1999). Although a subset of the sample was not asked to report experiences “prior to age 18, many of the items describe parents or stepparents in the household; therefore, responses are likely to reflect early life adversity. Cronbach’s alpha for the 8 categories in our study was 0.74.

#### 2.2.3 Subjective measure of sleep quality

The Pittsburgh Sleep Quality Index (PSQI) (Buysse et al., 1989), a 19-item self-report questionnaire, was used to assess subjective sleep quality over the past month. This scale is comprised of questions regarding 7 sleep components: subjective sleep quality, sleep latency, sleep duration, habitual sleep efficiency, sleep disturbances, use of sleeping medication, and daytime dysfunction. The global score, which is obtained by adding the seven component scores, can range from 0 to 21; and higher scores indicate poorer sleep quality. A global score greater than five is an indication of sleep impairment. Cronbach’s alpha for the 7 components was 0.65.

#### 2.2.4 Smoking and additional measures

Smoking history (average cigarettes per day, years of smoking), nicotine dependence levels using the Fagerström Test of Nicotine Dependence (FTND) (Heatherton et al., 1991), and exhaled carbon monoxide (CO) levels were collected. We also collected sociodemographic data (age, sex, ethnicity, marital status), as well as other biometric (Body Mass Index: BMI), psychological, and behavioral measures (alcohol and caffeine consumption).

### 2.3 Statistical analyses

Statistical analyses were performed using Statistical Package for the Social Sciences (SPSS) version 21.0 and Hayes’ PROCESS macro (version 3.1) for testing moderated mediation (Hayes, 2018).

#### 2.3.1 Primary analysis:

**Fig. 1:**
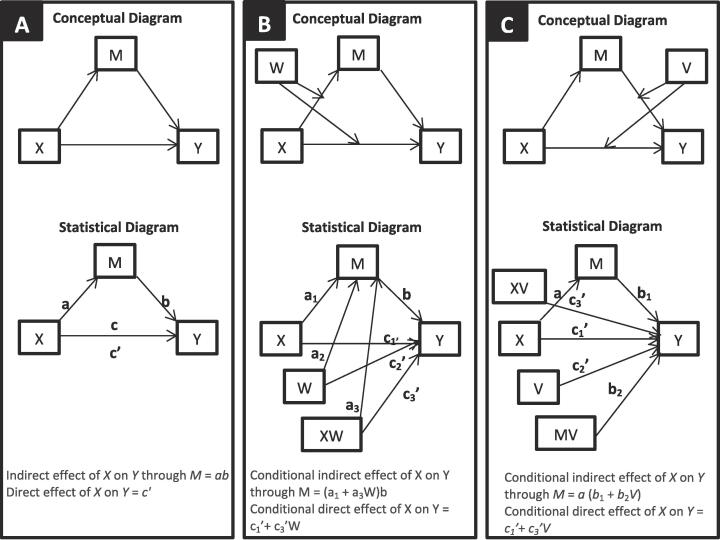
Simple mediation (A), first stage (B) and second stage (C) moderated mediation models.

We examined smoking status differences in sample characteristics using t-tests. Pearson’s correlations were used to test our first hypothesis and to examine the relationships among ELA, sleep variables, and depression symptoms. To test the second hypothesis and our research question, simple and moderated mediation models were used (Hayes, 2018) with the number of ELA as an independent variable (ACEs = X), depression symptoms as a dependent variable (PHQ9 score = Y), sleep quality (PSQI score = M) as a mediator, and smoking status as a moderator (W, V) (Fig. 1). The assumtion related to normal distribution of residuals was checked for each model. Models were run using Hayes’ PROCESS macro (Hayes, 2018) applying models 4 (simple mediation), 8 (first stage moderated mediation), and 15 (second stage moderated mediation), with 10,000 bias-corrected bootstrap samples. A p-value of 0.05 was set as the critical level of significance. Indices of the indirect effect and of moderated mediation were considered statistically significant if the 95% CI, estimated using bootstrap method, did not include zero.

#### 2.3.2 Secondary analysis

Additional exploratory mediation and moderated mediation analyses were performed to evaluate the indirect effect of ELA (independent variable) on depression (dependent variable) through each of the seven sleep components (mediator variables) captured by the PSQI, with smoking status as a moderator variable.

Models including age, sex, ethnicity, marital status, BMI, caffeine and alcohol consumption were also performed for each of the primary and secondary analyses.

## 3 Results

### 3.1 Sample characteristics

Participants ranged in age from 18 to 74 years (M = 35 years, SD = 12.6). The majority of the sample identified as White; and most were not married (see Table 1). On average, smokers smoked 15.6 cigarettes per day (SD = 6.9) for 11.0 years (SD = 10.3); and they had a baseline CO of 16.5 parts per million (SD = 11.3). The mean Fagerström Test for Nicotine Dependence (FTND) score was 5.3 (SD = 2.1), which indicates medium dependence on nicotine (Heatherton et al., 1991). The mean number of ACEs per participant was 2.1. Over 70% of the sample reported at least one ACE, and nearly one-quarter (23.7%) reported four or more ACEs. Overall, the current sample reported minimal depression symptoms (M = 2.3, SD = 3.2) and low sleep impairment, with a mean global PSQI score of 5 (SD = 3). Smokers had higher levels of depressive symptoms, impaired sleep quality, and ACE scores (Table 1).

**Table 1:** : Participant characteristics for the entire sample and by smoking status.

Variables	Full Sample			Smokers (n = 436)		Non-smokers (n = 141)		p-value
	**n (%)**	**mean (SD)**	**range**	**n (%)**	**mean (SD)**	**n (%)**	**mean (SD)**	
Age	578 (99.8)	35.00 (12.6)	18–74	436	35.30 (12.3)	141	34.20 (13.4)	0.35
Sex								
Female	262 (45.3)			188 (43.1)		74 (52.5)		0.05
Male	315 (54.4)			248 (56.9)		67 (47.5)		
Ethnicity								
White	421 (72.7)			323 (74.1)		98 (69.5)		0.25
Non-white	150 (25.9)			108 (24.8)		42 (29.80)		
Marital status								
Married	115 (19.9)			82 (18.8)		33 (23.4)		0.22
Single, Divorced, or Widowed	461 (79.6)			354 (81.2)		107 (75.9)		
BMI	563 (97.2)	26.8 (5.6)	17–60.5	424	27.05 (5.7)	138	26.20 (5.1)	0.1
Number of caffeinated drinks/day	505 (87.2)	1.15 (1.5)	0–12	376	1.3 (1.6)	128	0.65 (1)	**<0.001**
Alcohol consumption								0.4
Never	122 (21.1)			92 (21.1)		29 (20.6)		
Rarely	196 (33.9)			157 (36.0)		39 (27.7)		
Occasionally	105 (18.1)			74 (17.0)		31 (22.0)		
Sometimes	82 (14.2)			61 (14.0)		21 (14.9)		
Often	37 (6.4)			25 (5.7)		12 (8.5)		
Daily	29 (5.0)			21 (4.8)		8 (5.7)		
ACEs total score	578 (99.8)	2.10 (2.0)	0–8	435	2.30 (2.0)	141	1.46 (1.8)	**<0.001**
PHQ9	579 (1 0 0)	2.30 (3.2)	0–23	436	2.49 (3.0)	141	1.57 (2.0)	**<0.001**
PSQI global score	524 (90.5)	5.00 (3.0)	0–17	393	5.30 (3.0)	131	4.20 (2.0)	**<0.001**
Subjective sleep quality sub-score	558 (96.4)	0.85 (0.7)	0–3	418	0.90 (0.7)	140	0.69 (0.6)	**<0.001**
Sleep latency sub-score	554 (95.7)	1.12 (0.9)	0–3	417	1.20 (0.9)	137	0.88 (0.8)	**0.002**
Sleep duration sub-score	554 (95.7)	0.62 (0.8)	0–3	416	0.69 (0.8)	138	0.42 (0.6)	**<0.001**
Habitual sleep efficiency sub-score	544 (94.0)	0.39 (0.8)	0–3	409	0.45 (0.8)	135	0.21 (0.5)	**<0.001**
Sleep disturbances sub-score	539 (93.1)	1.16 (0.5)	0–3	403	1.16 (0.5)	136	1.15 (0.5)	0.70
Use of sleeping medication sub-score	558 (96.4)	0.21 (0.6)	0–3	418	0.22 (0.6)	140	0.15 (0.5)	0.17
Daytime dysfunction sub-score	558 (96.4)	0.73 (0.7)	0–3	418	0.74 (0.7)	140	0.71 (0.6)	0.75

SD = Standard Deviation; BMI: Body Mass Index; ACEs = Adverse Childhood Experiences; PHQ9 = Patient Health Questionnaire- 9; PSQI = Pittsburgh Sleep Quality Index.

### 3.2 Primary analyses

**Fig. 2:**
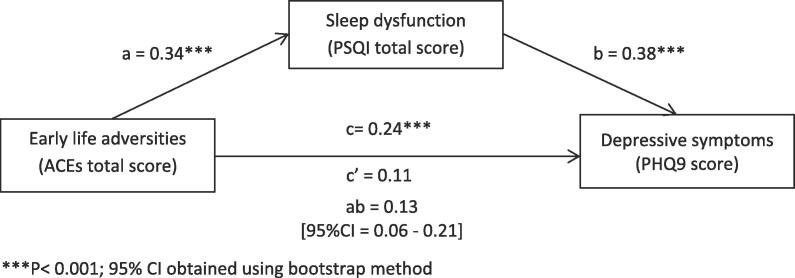
Simple mediation model: Indirect effect of early life adversities on depression through sleep dysfunction (n = 523).

Depression symptoms were significantly, positively associated with the number of ACEs experienced by the participants, which provides support for the first hypothesis (r = 0.11, p < 0.01). In addition, sleep quality was significantly, positively correlated with the number of ACEs (r = 0.23; p < 0.001) and with depression symptoms (r = 0.36; p < 0.001) (Table 2). Consistent with our second hypothesis, sleep quality fully mediated the relationship between the number of ACEs and depressive symptoms (Fig. 2). Two further models (first stage and second stage moderated mediation) were tested to assess the moderating role of smoking in this mediation. Only the second stage analysis was significant, index = 0.10, SE = 0.04, 95%CI [0.03; 0.19], indicating that the indirect effect of ELA on depression symptoms through their effect on sleep quality is stronger among smokers, b = 0.14, SE = 0.04, 95%CI [0.07; 0.23], than among non-smokers, b = 0.04, SE = 0.02; 95%CI [0.0002; 0.10] (Table 3). Similar results were found for mediation and moderated mediation models when adjusting for age, sex, ethnicity, marital status, BMI, caffeine, and alcohol consumption (supplementary Fig. 1 and Table 1).

**Table 2:** Pearson’s correlations among relevant study variables.

	ACEs	PSQI	PHQ9	Smoking status	Age	Sex†	Ethnicity††	Marital††† status
ACEs	1	0.23***(n = 523)	0.11**(n = 578)	0.18***(n = 576)	−0.01(n = 577)	−0.08(n = 576)	0.11**(n = 571)	0.01(n = 560)
PSQI		1	0.36***(n = 524)	0.17***(n = 524)	−0.07(n = 524)	−0.04(n = 524)	0.05(n = 518)	−0.08(n = 507)
PHQ9			1	0.12**(n = 577)	−0.13**(n = 578)	−0.02(n = 577)	0.09*(n = 571)	−0.16***(n = 560)
Smoking status				1	0.04(n = 577)	0.08(n = 577)	−0.05(n = 571)	−0.05(n = 559)
Age					1	0.01(n = 577)	−0.03(n = 571)	0.30***(n = 560)
Sex						1	0.01(n = 571)	−0.08(n = 559)
Ethnicity							1	0.10*(n = 554)
Marital status								1

*p < 0.05; **<0.01; ***p < 0.001

Sex†: male = 1 vs female = 0; Ethnicity††: non-white = 1 vs white = 0; Marital††† status: married = 1 vs (single, divorced, or widowed) = 0

ACEs = Adverse Childhood Experiences; PSQI = Pittsburgh Sleep Quality Index; PHQ9 = Patient Health Questionnaire- 9

**Table 3:** Moderated mediation models results (n = 523).

Model 2.a (1 st stage)	β	SE	t	p	95% LLCI	95% ULCI
X --> M (a_1_)	0.22	0.14	1.60	0.11	−0.05	0.50
W --> M (a_2_)	0.90	0.30		0.07	−0.07	1.42
X * W --> M (a_3_)	0.10	0.16	0.67	0.50	−0.20	0.41
M --> Y (b)	0.37	0.05	7.80	<0.001	0.28	0.46
X --> Y (c’_1_)	−0.003	0.15	−0.02	0.98	−0.30	0.30
W -->Y (c’_2_)	0.40	0.41	0.97	0.33	−0.41	1.21
X*W -->Y (c’_3_)	0.11	0.17	0.68	0.50	−0.22	0.45
Conditional direct effect for smokers	0.11	0.08	1.40	0.16	−0.04	0.27
Conditional direct effect for non-smokers	−0.003	0.15	−0.02	0.98	−0.30	0.30
	**β**	**Boot SE**			**Boot 95% LLCI**	**Boot 95% ULCI**
Conditional Indirect effect for smokers	0.12	0.04			0.06	0.20
Conditional Indirect effect for non-smokers	0.08	0.04			0.01	0.17
Index of moderated mediation	0.04	0.05			−0.06	0.14
Model 2.b (2nd stage)	**β**	**SE**	**t**	**p**	**95% LLCI**	**95% ULCI**
X --> M (a)	0.34	0.06	5.47	<0.001	0.22	0.47
M --> Y (b_1_)	0.12	0.12	0.99	0.32	−0.12	0.35
M*V -->Y (b_2_)	0.29	0.13	2.26	0.02	0.04	0.55
X --> Y (c’_1_)	0.05	0.15	0.35	0.73	−0.24	0.35
V -->Y (c’_2_)	−0.80	0.66	−1.17	0.24	−2.08	0.52
X*V -->Y (c’_3_)	0.04	0.17	0.26	0.80	−0.29	0.38
Conditional direct effect for smokers	0.10	0.08	1.22	0.22	−0.06	0.25
Conditional direct effect for non-smokers	0.05	0.15	0.35	0.73	−0.24	0.35
	**Β**	**Boot SE**			**Boot 95% LLCI**	**Boot 95% ULCI**
Conditional Indirect effect for smokers	0.14	0.04			0.07	0.23
Conditional Indirect effect for non-smokers	0.04	0.02			0.0002	0.10
Index of moderated mediation	0.10	0.04			0.03	0.19

Notes: X = ACEs total score; Y = Depressive symptoms assessed by PHQ9 score; M = Sleep quality assessed by PSQI global score [Mediator]; W/V = Smoking status [Moderator].

Boot SE: Bootstrapped standard errors; Boot 95%LLCI and Boot 95% ULCI: 95% bootstrapped confidence intervals

### 3.3 Secondary analyses

Correlations for each of the sleep components with ELA, depression symptoms, and smoking status are summarized in Table 4. Subsequent mediation analyses were run separately for each sleep component if assumptions related to significant correlations between variables in the models were met. Thus, mediation analyses were performed for five of the PSQI components; subjective sleep quality (Table 5: model 3), sleep latency (Table 5: model 4), sleep duration (Table 5: model 5), sleep disturbances (Table 5: model 6), and daytime dysfunction (Table 5: model 7). Each of these five components mediated partially the relationship between ELA and depression. Furthermore, there was evidence of moderated mediation for two of the five components: sleep latency (index = 0.06, SE = 0.02, 95%CI [0.01, 0.12]) and sleep duration (index = 0.06, SE = 0.03, 95%CI [0.01, 0.13]) (Table 5: models 8 and 9 respectively). For these two components, significant conditional indirect effects of ELA on depression through the sleep components were found only among smokers. Similar results were found for adjusted moderated mediation models (supplementary Table 2).

**Table 4:** Correlations among early life adversities, depression, smoking status, and sleep variables.

	ELA†	Depression symptoms	Smoking status
Subjective sleep quality	0.18***(n = 557)	0.27***(n = 558)	0.13**(n = 558)
Sleep latency	0.16***(n = 553)	0.27***(n = 554)	0.14**(n = 554)
Sleep duration	0.13**(n = 553)	0.18***(n = 554)	0.15***(n = 554)
Habitual sleep efficiency	0.07(n = 543)	0.17***(n = 554)	0.13**(n = 554)
Sleep disturbances	0.24***(n = 538)	0.20***(n = 539)	0.01(n = 539)
Use of sleeping medication	0.02(n = 557)	0.10*(n = 558)	0.05(n = 558)
Daytime dysfunction	0.12**(n = 557)	0.25***(n = 558)	0.01(n = 558)

Notes: * p < 0.05; ** p < 0.01; ***p < 0.001;

†ELA = Early Life Adversities

**Table 5:** Mediation and second stage moderated mediation models, secondary analysis.

Model 3 : M = subjective sleep quality (n = 557)	β	p	95% LLCI	95% ULCI
X --> Y (c’)	0.13	0.05	−0.001	0.26
X --> M -->Y (a*b)	0.07		0.03	0.13 †
Model 4 : M = sleep latency (n = 553)	**β**	**p**	**95% LLCI**	**95% ULCI**
X --> Y (c’)	0.15	0.03	0.02	0.28
X --> M -->Y (a*b)	0.07		0.03	0.12 †
Model 5 : M = sleep duration (n = 553)	**β**	**p**	**95% LLCI**	**95% ULCI**
X --> Y (c’)	0.17	0.01	0.04	0.31
X --> M -->Y (a*b)	0.03		0.01	0.07 †
Model 6 : M = sleep disturbances (n = 538)	**β**	**p**	**95% LLCI**	**95% ULCI**
X --> Y (c’)	0.15	0.03	0.02	0.30
X --> M -->Y (a*b)	0.07		0.03	0.13 †
Model 7: M = daytime dysfunction (n = 557)	**β**	**p**	**95% LLCI**	**95% ULCI**
X --> Y (c’)	0.16	0.02	0.02	0.29
X --> M -->Y (a*b)	0.05		0.01	0.09 †
Model 8 : M = sleep latency (n = 553)	**β**		**Boot 95% LLCI**	**Boot 95% ULCI**
Conditional indirect effect for non-smokers	0.02		−0.01	0.05
Conditional indirect effect for smokers	0.08		0.03	0.13
Index of moderated mediation	0.06		0.01	0.12
Model 9 : M = sleep duration (n = 553)	**β**		**Boot 95% LLCI**	**Boot 95% ULCI**
Conditional indirect effect for non-smokers	−0.02		−0.06	0.003
Conditional indirect effect for smokers	0.04		0.01	0.08
Index of moderated mediation	0.06		0.01	0.13

Notes: X = ACEs total score; Y = Depressive symptoms assessed by PHQ9 score.

Boot 95%LLCI and Boot 95% ULCI: 95% bootstrapped confidence intervals.

† 95% CI estimated using bootstrap method.

## 4 Discussion

Results of this study demonstrate that ELA and depression symptoms are positively associated; and sleep quality fully mediates this relationship. These findings are consistent with Jones et al. (Jones et al., 2018), and they extend previous research linking a range of adverse experiences in childhood to depression through their association with different mediators (Liu, 2017)**.** This investigation is one of the few that has identified sleep as a potential mediator through which ELA exerts a depressogenic effect. In addition to being the first to document this mediation effect in a broader clinical population, to our knowledge, this is the first examination of this set of linkages in tobacco smokers.

The associations of sleep quality with depression and ELA are well established (Kajeepeta et al., 2015, Fang et al., 2019), as is the role of sleep as an important regulator of circadian systems and as a promoter of homeostasis and environmental adaptation (Deboer, 2018). Therefore, the mediating effect of sleep quality in the relationship between ELA and depression is not surprising. Sleep problems occurring after trauma exposure and the resulting chronodisruption, may alter the fundamental properties of brain systems regulating neuroendocrine, immune, and autonomic function; and these changes may play a crucial role in the development of stress related disorders and ELA-related comorbidities, such as depression, through impaired homeostatic balance (Morris et al., 2012, Agorastos et al., 2019, Wulff et al., 2010). The therapeutic benefit of sleep management on depression further supports the strong mechanistic links between these factors (Tchekalarova et al., 2018, Perera et al., 2016, Zhao et al., 2018, Isaac and Greenwood, 2011).

In addition to extending previous research on the relationship between ELA and depression by evaluating the mediating effect of sleep, this study sheds light on the moderating role of tobacco use in these relationships. The influence of tobacco use is novel and of significance in the context of the diverse impacts that smoking has on both sleep and depression. Our finding that tobacco use moderates the mediating relationship of sleep quality in predicting depression may be related to the ongoing and chronic effects of tobacco on various neurobiological processes, including hormonal (al'Absi et al., 2003, al'Absi et al., 2004) and stress-related systems (al'Absi et al., 2013) as well as sleep regulation processes (Jaehne et al., 2009). Such dysregulation may contribute directly or indirectly to the risk for depression (Mathew et al., 2017), enhancing the expected indirect effect of ELA on depression via sleep. While we treated smoking as a dichotomous moderator in the current study due to its chronic, exogenous pharmacological effects on sleep and depression, future studies might consider alternative roles or measures of smoking behavior in modeling the relationship between ELA and depression.

Our analysis of specific sleep components of PSQI revealed that subjective sleep quality, sleep latency, sleep duration, sleep disturbances, and daytime dysfunction were associated with ELA and depression. These results were consistent with previous studies that identified a wide array of sleep problems related to depression (Liu et al., 2018, Benca and Peterson, 2008) and to ELA among general (Kajeepeta et al., 2015, Koskenvuo et al., 2010, Chapman et al., 2011, Greenfield et al., 2011) and psychiatric populations (Hamilton et al., 2018). Unlike Greenfield and colleagues (Greenfield et al., 2011), however, we did not find evidence that sleep efficiency nor use of sleep medication were significantly related to ELA. Furthermore, the results from our secondary analyses suggest that the stronger indirect effect of ELA on depression via sleep quality that we found among smokers compared to non-smokers may be attributable primarily to greater delays in sleep onset and to shorter sleep duration among smokers. The observation of mediating effects for sleep onset and sleep duration only among smokers is novel and worth further examination in prospective studies.

We note, here, some limitations in this study. First, there are potential recall biases influencing responses on the ACE questionnaire; and it is possible that current depression symptoms influence perceptions of adversities. Second, we used ACE total scores that represented overall exposure to eight categories of ELA; and a subset of our sample may have included some experiences after age 18. We did adjust for the different instructions in ancillary analyses and found no effects of the different versions of instruction on the reported findings, which confirms that the observed relationships are likely to be linked to life adversity independent of specific time of exposure. Future research should further examine this role of age of exposure in the observed relationships and whether the relationships differ across types or categories of adversities. For example, being sexually abused could have different psychological and behavioral implications than having a household with mental illness and/or substance abuse. Future research should improve assessment of life adversity to include frequency of exposure, intensity and duration of exposure, and age of exposure. Third this study did not include an objective assessment of sleep quality. Finally, as with all cross-sectional studies, this study cannot rule-out alternative causal directions in the relationships among ELA, sleep disturbances, and depression. Despite these limitations, the current study provides evidence consistent with a mediating pathway linking ELA to depression through sleep quality; and our exploratory analyses identify promising targets for clinical intervention. Our results suggest that individuals who experience ELA, who have sleep problems, and who are smokers may be considered at high risk of experiencing depression symptoms and should be routinely screened for early diagnosis and treatment. Evaluation of personalized treatment protocols targeting sleep-related consequences of ELA should be considered in future research, given poor treatment outcomes for depression among adults with a history of childhood adversities (Nanni et al., 2012, Klein et al., 2009). Furthermore, early detection of sleep problems among smokers who have experienced high levels of adversities as well as control of these modifiable risk factors may be effective ways to break the risk pathway linking ELA to depression.

## 5 Conclusion

The current study proposed a model in which sleep quality is a mediator of the ELA –depression relationship, with smoking as a potential moderator of this mediation. Promising targets for intervention are suggested based on model findings; and further studies are needed to provide evidence of the utility of such models in treatment.

## Funding

This research was supported in part by grants from the National Institute of Health (R01DA016351 and R01DA027232).

## Declaration of Competing Interest

The authors declare that they have no known competing financial interests or personal relationships that could have appeared to influence the work reported in this paper.
