# Supplementary material for: Effects of tobacco addiction on links between early life adversities, sleep disturbance, and depression: A moderated mediation approach
Source: Prev Med Rep. 2020 Oct 20;20:101225. doi: 10.1016/j.pmedr.2020.101225 (PMC7642869; doi:10.1016/j.pmedr.2020.101225)
Supplement: Supplementary data 1 [file mmc1.docx]

Supplementary Table 1: Moderated mediation adjusted models *

| Model 2.a (1 ^st^ stage) | β | SE | t | p | 95% LLCI | 95% ULCI |
| --- | --- | --- | --- | --- | --- | --- |
| X --> M (a_1_) | 0.24 | 0.16 | 1.56 | 0.12 | -0.06 | 0.55 |
| W--> M (a_2_) | 0.76 | 0.43 | 1.76 | 0.08 | -0.09 | 1.60 |
| X * W --> M (a_3_) | 0.10 | 0.17 | 0.60 | 0.55 | -0.24 | 0.45 |
| M --> Y (b) | 0.28 | 0.05 | 5.7 | <0.001 | 0.18 | 0.38 |
| X --> Y (c’_1_) | 0.08 | 0.15 | 0.52 | 0.60 | -0.23 | 0.39 |
| W-->Y (c’_2_) | 0.67 | 0.43 | 1.54 | 0.12 | -0.18 | 1.53 |
| X*W-->Y (c’_3_) | -0.03 | 0.18 | -0.17 | 0.87 | -0.38 | 0.32 |
| Conditional direct effect for smokers | 0.05 | 0.08 | 0.61 | 0.54 | -0.11 | 0.22 |
| Conditional direct effect for non-smokers | 0.08 | 0.16 | 0.51 | 0.60 | -0.23 | 0.39 |
|  | **β** | **Boot SE** |  |  | **Boot 95% LLCI** | **Boot 95% ULCI** |
| Conditional Indirect effect for smokers | 0.10 | 0.04 |  |  | 0.04 | 0.18 |
| Conditional Indirect effect for non-smokers | 0.07 | 0.04 |  |  | 0.01 | 0.16 |
| Index of moderated mediation | 0.03 | 0.04 |  |  | -0.06 | 0.12 |
| Model 2.b (2^nd^ stage) | **β** | **SE** | **t** | **p** | **95% LLCI** | **95% ULCI** |
| X --> M (a) | 0.37 | 0.07 | 5.1 | <0.001 | 0.22 | 0.51 |
| M --> Y (b_1_) | 0.03 | 0.12 | 0.28 | 0.78 | -0.20 | 0.27 |
| M*V -->Y (b_2_) | 0.29 | 0.13 | 2.23 | 0.03 | 0.03 | 0.55 |
| X --> Y (c’_1_) | 0.15 | 0.16 | 0.95 | 0.34 | -0.16 | 0.47 |
| V-->Y (c’_2_) | -0.50 | 0.68 | -0.73 | 0.47 | -1.84 | 0.85 |
| X*V -->Y (c’_3_) | -0.12 | 0.18 | -0.64 | 0.52 | -0.47 | 0.24 |
| Conditional direct effect for smokers | 0.04 | 0.08 | 0.42 | 0.67 | -0.13 | 0.20 |
| Conditional direct effect for non-smokers | 0.15 | 0.16 | 0.95 | 0.34 | -0.16 | 0.47 |
|  | **Β** | **Boot SE** |  |  | **Boot 95% LLCI** | **Boot 95% ULCI** |
| Conditional Indirect effect for smokers | 0.12 | 0.04 |  |  | 0.05 | 0.21 |
| Conditional Indirect effect for non-smokers | 0.01 | 0.03 |  |  | -0.03 | 0.07 |
| Index of moderated mediation | 0.11 | 0.04 |  |  | 0.03 | 0.21 |

*Models adjusted for age, sex, ethnicity, marital status, BMI, caffeine consumption, alcohol consumption.

Notes: X = ACEs total score; Y = Depressive symptoms assessed by PHQ9 score; M = Sleep quality assessed by PSQI global score [Mediator]; W/V = Smoking status [Moderator].

Boot SE: Bootstrapped standard errors; Boot 95%LLCI and Boot 95% ULCI: 95% bootstrapped confidence intervals

Supplementary Table 2: Moderated mediation adjusted models for sleep latency and sleep duration as mediators*

|  | β |  | Boot 95% LLCI | Boot 95% ULCI |
| --- | --- | --- | --- | --- |
| Mediator= sleep latency |  |  |  |  |
| Conditional indirect effect for non-smokers | 0.01 |  | -0.01 | 0.05 |
| Conditional indirect effect for smokers | 0.06 |  | 0.02 | 0.12 |
| Index of moderated mediation | 0.05 |  | 0.01 | 0.12 |
| Mediator= sleep duration |  |  |  |  |
| Conditional indirect effect for non-smokers | -0.03 |  | -0.08 | 0.008 |
| Conditional indirect effect for smokers | 0.04 |  | 0.007 | 0.09 |
| Index of moderated mediation | 0.07 |  | 0.01 | 0.16 |

*Models adjusted for age, sex, ethnicity, marital status, BMI, caffeine consumption, alcohol consumption.

Notes: Boot 95%LLCI and Boot 95% ULCI: 95% bootstrapped confidence intervals

c = 0.19**

Early life adversities (ACEs total score)

Depressive symptoms

(PHQ9 score)

Sleep dysfunction (PSQI total score)

a =0.37***

b = 0.29***

c’ = 0.08

ab = 0.11

[95%CI = 0.06 - 0.21]

***P< 0.001; **P<0.05; 95% CI obtained using bootstrap method

Supplementary Figure 1: Simple mediation model: Indirect effect of early life adversities on depression through sleep dysfunction adjusted for age, sex, ethnicity, marital status, BMI, caffeine consumption, alcohol consumption, n= 433.
